# Cellular senescence-related genes: predicting prognosis in hepatocellular carcinoma

**DOI:** 10.1186/s12885-023-11288-1

**Published:** 2023-10-18

**Authors:** Weiwei Yuan, Yuanmin Xu, Zhiheng Wu, Yang Huang, Lei Meng, Shiping Dai, Songcheng Ying, Zhangming Chen, Aman Xu

**Affiliations:** 1 Department of General Surgery, Anhui Public Health Clinical Center, The First Affiliated Hospital of Anhui Medical University, Hefei, 230012 China; 2 Department of General Surgery, First Affiliated Hospital of Anhui Medical University, Hefei, 230022 China; 3 Department of General Surgery, Wuwei City People’s Hospital, Wuhu, 241000 China; 4 Department of Immunology, School of Basic Medical Sciences, Anhui Medical University, Hefei, 230032 China

**Keywords:** Cellular Senescence, Immunotherapy, Hepatocellular Carcinoma (HCC), Biomarkers, Chemotherapy

## Abstract

Recent studies have shown that the high incidence and low cure rate of hepatocellular carcinoma (HCC) have not improved significantly. Surgery and liver transplantation are the mainstays of prolonging the survival of HCC patients. However, the surgical resection rate of HCC patients is very low, and even after radical surgical resection, the recurrence rate at 5 years postoperatively remains high and the prognosis is very poor, so more treatment options are urgently needed. Increasing evidence suggests that cellular senescence is not only related to cancer development but may also be one of its primary driving factors. We aimed to establish a prognostic signature of senescence-associated genes to predict the prognosis and therapeutic response of HCC patients. The aim of this study was to develop a risk model associated with cellular senescence and to search for potential strategies to treat HCC. We divided HCC patients into two clusters and identified differentially expressed genes (DEGs) between clusters. In this study, low-risk patients had a better prognosis, higher levels of immune cell infiltration, and better efficacy to fluorouracil, Paclitaxel and Cytarabine chemotherapy compared to high-risk patients. To further identify potential biomarkers for HCC, we further validated the expression levels of the four signature genes in HCC and neighbouring normal tissues by in vitro experiments. In conclusion, we identified and constructed a relevant prognostic signature, which performed well in predicting the survival and treatment response of HCC patients. This helps to differentiate between low-score and high-risk HCC, and the results may contribute to precise treatment protocols in clinical practice.

**Supplementary Information:**

The online version contains supplementary material available at 10.1186/s12885-023-11288-1.

## Introduction

HCC ranks at the third leading cause of death due to cancer worldwide and is one of the most prevalent liver malignancies, posing a threat to human health [1]. According to global cancer statistics, the mortality rate of HCC in 2020 was 8.3% [2]. HCC ranks fifth in global incidence and second highest in male mortality [3]. In China, the key determinants were chronic HBV infection, aflatoxin exposure, or both [4, 5]. For the treatment of HCC, the early stage is treated by surgery. However, in China, more than 80% of patients are already in advanced stages of cancer at the time of diagnosis and have lost the opportunity for surgery. With the progress of treatment for advanced HCC, the overall survival(OS)and progression-free survival (PFS) have been continuously improved [6, 7]. Nowadays, with the application of tumor immunotherapy and immune checkpoint inhibitors, the treatment of tumors has made continuous progress. Immunotherapy can be used in the treatment of many kinds of malignant tumours to restore and improve the anti-tumour ability of the patient’s immune system, and it has fewer side effects compared with chemotherapy [8, 9]. However, the biggest limitation of immunotherapy at present is its low efficiency, the efficiency of PD-1 is only 15–50% [10, 11], drug resistance is difficult to overcome, currently it is only used for adjuvant therapy [12], so we have to actively search for new potential biomarkers, vaccines for immunotherapy and immune checkpoint inhibitors.

Cellular senescence is a fundamental cellular response procedure that is triggered upon the application of certain specific stimuli to the cell. Once cellular senescence occurs, cell proliferation irreversibly stops. The main hallmark of cellular senescence is the cessation of the cell division cycle and the expression of senescence-specific markers Aging-related-β-galactosidase (SA-β-Gal) [13]. Cellular senescence can act as an inherent cytoprotective mechanism against external stress stimuli ,which also plays a role in limiting tumor progression [14]. If a large number of senescence genes can be identified and made potential targets for cancer in clinical treatment, tumor growth inhibition by inducing tumor cells to reacquire senescence may become a reality. Therefore, studies on cellular senescence in HCC are necessary.

Many studies are now applying genetic prognostic models based on the TCGA database to various cancers. Currently, few studies have focused on the diagnosis, survival and prognosis of cellular senescence-related genes in HCC. Therefore, our study aimed to construct a senescence-related network that showed significant validity in predicting patient survival, immune cell infiltration status, immune checkpoint gene expression and sensitivity to chemotherapeutic agents. We constructed a cellular senescence prognostic risk score signature by analyzing the role of cellular senescence genes and cellular senescence differential genes in HCC. This signature not only independently predicts the prognosis of HCC patients and the clinical characteristics of patients, but also effectively differentiates patients who are more sensitive to chemotherapeutic agents and immunotherapy. The results of this study may provide new tactics to explore the treatment of HCC.

## Materials and methods

### Transcriptome data download and access to clinical information

Transcriptomic data from 374 tumor tissues and 50 normal tissues of HCC patients were first downloaded and collated from The Cancer Genome Atlas (TCGA) website (https://portal.gdc.cancer.gov), followed by downloading clinical data of HCC patients and merging the data. The gene symbol ID was translated to gene name in transcriptome data. The downloaded data were collated using Perl software (v5.30.0) and R software (v4.1.2). We downloaded 279 genes associated with cellular senescence from the CellAge website (Supplementary Table S1). Age, sex, grading and pathological TNM staging were extracted from the clinical data and combined. Incomplete clinical information was excluded from our study.

### Visualization of differential genes

Differential analysis of senescence-related genes was performed using the “limma” package of R software to visualize differentially expressed genes (DEG) in normal and tumor samples in HCC. The optimal cut-off points for identifying DEG were set as *P* value < 0.05 and |logFC (fold change)| > 1. Heat maps and volcano maps of DEG were produced.

### Interaction network between proteins

The online STRING database was used to construct the protein interaction network of DEG (http://string-db.org). Network visualization of the association of proteins encoded by differential senescence genes (interaction score > 0.40), limited to “Homo sapiens”. Data files were imported into Cytoscape software for visual editing (https://cytoscape.org/).

### Construct and validate a risk scoring methodology

We divided the HCC data into a training cohort, which was used to construct the prognostic model, and a test cohort, which was used to validate the accuracy of the prognostic model values for aging-related genes. Risk score formula: risk score = ∑i1 (Coefi∗ExpGenei).“ Coef,“ regression coefficient; “ExpGene,“ gene expression. Samples were classified into high and low risk groups based on the median value of risk score for each sample. Prognosis-related false-positive aging-related genes were first eliminated by LASSO Cox regression analysis, and the set of genes corresponding to the point with the smallest error was found to be the characteristic genes of the model by cross-validation. The prognosis-related senescence-associated genes were then evaluated by a multifactorial Cox regression analysis model on OS and clinical outcomes of HCC patients. Finally, the predicted independent prognostic gene sets were used to further construct the prognostic model. Kaplan-Meier curves were plotted using the “survival” and “monitor” packages to analyze survival differences (including 5-year survival) between the two cohorts. ROC curves were plotted with the “timeROC” package to predict the predictive accuracy of the two cohort characteristics.

### Evaluation and validation of prognostic models

We included risk score and four clinicopathological factors, namely age, gender, grade and stage, in univariate and multivariate Cox regression analyses to verify whether risk score and these four factors could be independent predictors of prognosis in patients with HCC. *p* < 0.05 was considered significant. Time-dependent ROC curves were used to assess the ability of the risk score to predict overall survival (OS). We also used the C-index to compare differences in the efficiency of risk scores and clinicopathological factors in predicting the prognosis of HCC patients. To facilitate individualized assessment of each case, factors with significant outcomes in multivariate analysis were used to construct the nomogram. Calibration curves were generated to assess whether the predicted patient (1-year, 3-year, 5-year) survival probabilities of the nomogram were close to the true probabilities. Principal component analysis (PCA) was performed using the ggplot2 R package to see if patients could be classified into different categories based on the amount of senescence-related gene expression. In turn, the prognostic significance of different clinicopathological factors on HCC patients was assessed.

### Gene set enrichment analysis

To identify enrichment pathways associated with the risk score, GSEA software (v4.2.1) and gene set (C2. Cp.kegg. V7.4. Symbols GMT) were used to conduct gene set enrichment analysis (GSEA) for the high- and low-risk groups. According to the criteria of FDR < 0.05 and *p* < 0.05, the top-ranked pathways in the high-risk and low-risk groups were selected.

### Immunoassay and immunotherapy prediction

To analyze the relationship between immune cell content and risk score, we downloaded immune cell infiltration files for all TCGA tumors from the TIMER 2.0 website (http://timer.cistrome.org/). Perform Limma, scales, ggplot2, ggtext R packages to generate bubble plots to show the correlation between immune cell content and risk scores. The limma R software package was used to analyze the difference in immune cell content between the high-risk and low-risk groups and was visualized by generating a heat map using the pheatmap R software package. Immune cell difference analysis (ssGSEA) was used to evaluate the degree of tumor immune infiltration in different types. The differences in immune cells and immune function between the high-risk group and the low-risk group were shown by Wilcoxon signature rank test with boxplots. We used the ESTIMATE algorithm to calculate the immune and stromal scores in HCC to predict the content of immune and stromal cells. In addition, we compared the immune checkpoint expression between the high-risk and low-risk groups by the Wilcoxon signed rank test.

### Human protein atlas (HPA)

In HPA (https://www.proteinatlas.org/), sections from microarrays of cancerous tissues can be obtained for immunohistochemical staining and the corresponding slides scanned to generate digital images. We obtained immunohistochemical staining images of different proteins EZH2,G6PD,LGALS3 and PSMD14 in HCC and normal tissues from the HPA website.

### Quantitative real-time PCR

Paired cancer tissue samples from 10 HCC patients were collected from the Department of General Surgery, The First Affiliated Hospital of Anhui Medical University. All patients or authorized relatives signed the informed consent, and the experimental design met the requirements of the ethics committee. All patients had no preoperative neoadjuvant chemotherapy and no previous immune-related diseases. TRIzol reagent was used to extract and prepare the total RNA extract from the tissue, which was reversely transcribed into cDNA for qRT-PCR. After activating the CT value of the target sample, the relative expression level of the target gene was calculated by 2 − ΔΔCt with the adjacent tissue as the control. Human GAPDH was used as an internal reference. Differences in the expression of four pairs of senescence-related genes between hepatocellular carcinoma tissues and adjacent non-cancerous tissues were tested by t-test. Use the GraphPad Prism 8.0 software to draw the graphs. The primer sequences used in this study are listed in (Supplementary Table S2).

### Statistical data analysis

We use R 4.1.0 software to analyze the data, and use Strawberry Perl-5.32.1.1 to run the script in the script analysis. Normal distribution was analyzed using Student ‘s t-test. Non-normal distribution parameters were tested by Wilcoxon rank sum test. Statistical data were analyzed by Pearson chi-square test. * *p* < 0.05 was considered statistically significant, * * *p* < 0.01 was considered statistically significant, * * * *p* < 0.001 was considered statistically significant.

## Results

### Study workflow

**Fig. 1:**
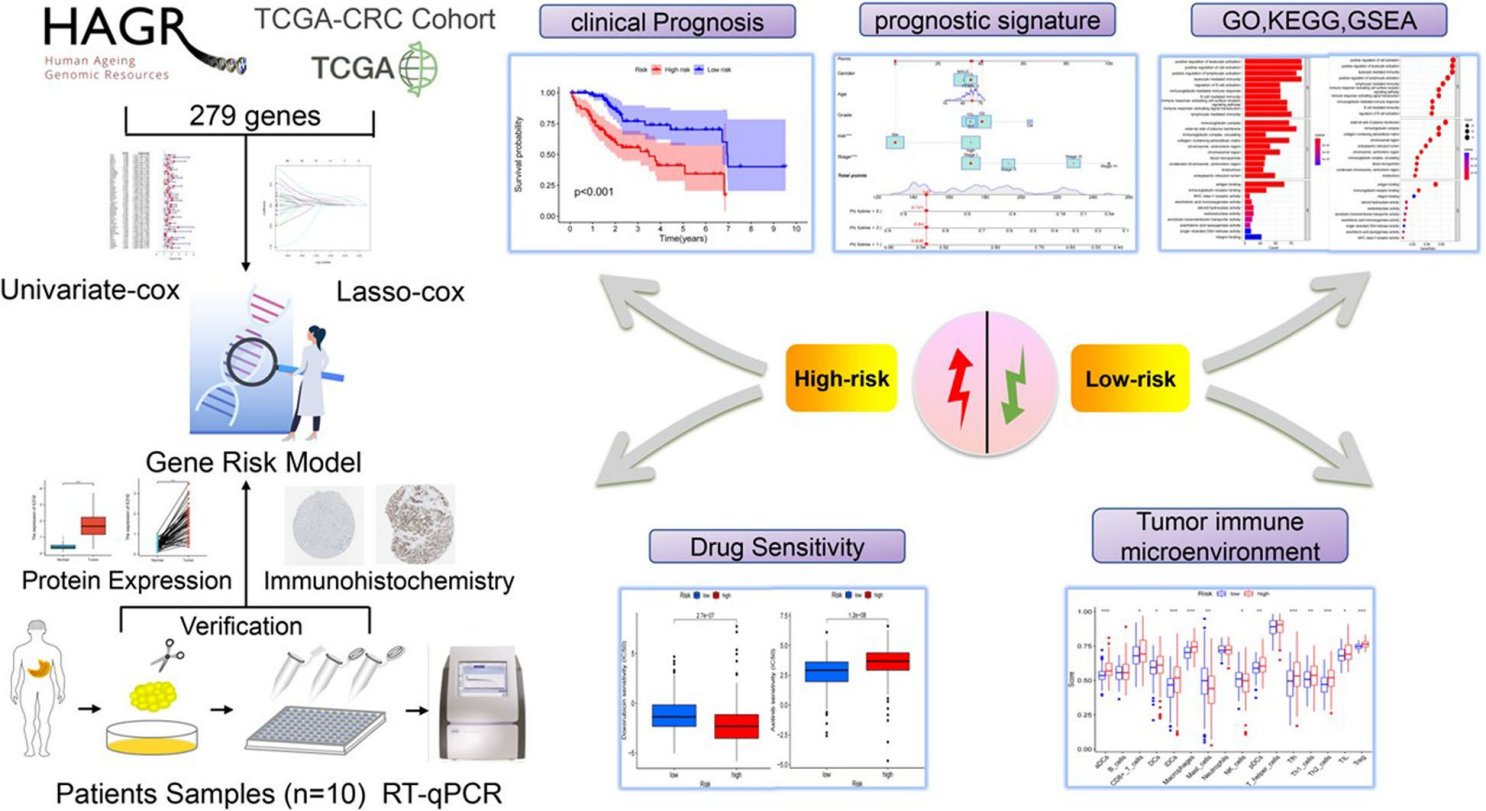
Detailed flow chart of the research

Flowchart of this study, we used an integrated bioinformatics approach, functional analysis and some experimental validation in the article (Fig. 1).

### Identification of differentially expressed cellular senescence-associated genes

**Fig. 2:**
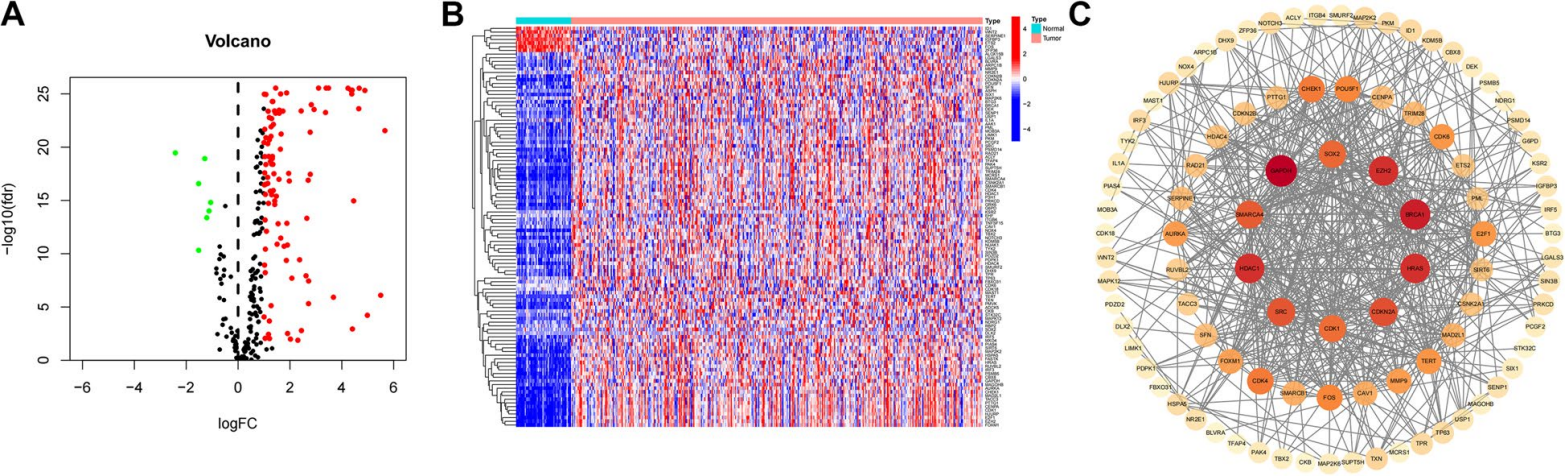
Identification of differentially expressed cellular senescence-associated genes. Heat map (**A**) and volcano map (**B**) for differential analysis of senescence-related genes in tumor samples and normal tissues in HCC identified using TCGA. Network circle diagram of senescence-related genes protein interactions **C**

In the TCGA database, we downloaded the expression and clinical information of 252 HCC patients. By comparing the differential expression of senescence-related genes in gastric cancer tissues and cancer-adjacent normal tissues (logFCfilter = 1, fdrFilter < 0.05), we identified 109 DEGs, which were plotted as volcano plots (Fig. 2A) and heat maps (Fig. 2B), where the red dots are the up-regulated genes, and the green dots are the down-regulated genes. The horizontal axis is Log2 (fold change), the more off-centre the point is, the greater the fold difference is; the vertical axis is -Log 10 (adjusted *P*-value), the more upward the point is, the more significant the difference is. Compared with the down-regulated genes in green, the up-regulated genes in red have more significant differences and larger fold differences. Next, to screen for DEGS-related interacting proteins, we imported 109 DEGS into the STRING website using the STRING database and mapped gene networks and protein-protein interaction networks (interaction scores greater than 0.40) that may interact with senescence-related genes. Network maps of direct and indirect effects of senescence-related genes were generated by removing disconnected nodes and then importing the data into the Cytoscape software for visualisation and editing.The core functional modules of the PPI network were obtained from the MCODE plug-in of the Cytoscape software consisting of 10 genes, including (SOX2,E2H2,BRCA1,HRAS, CDKN2A, CDK1, SRC, HDAC1, GAPDH and SMARCA4) (Fig. 2C).

### Prognostic characterization of differential genes in cellular senescence

**Fig. 3:**
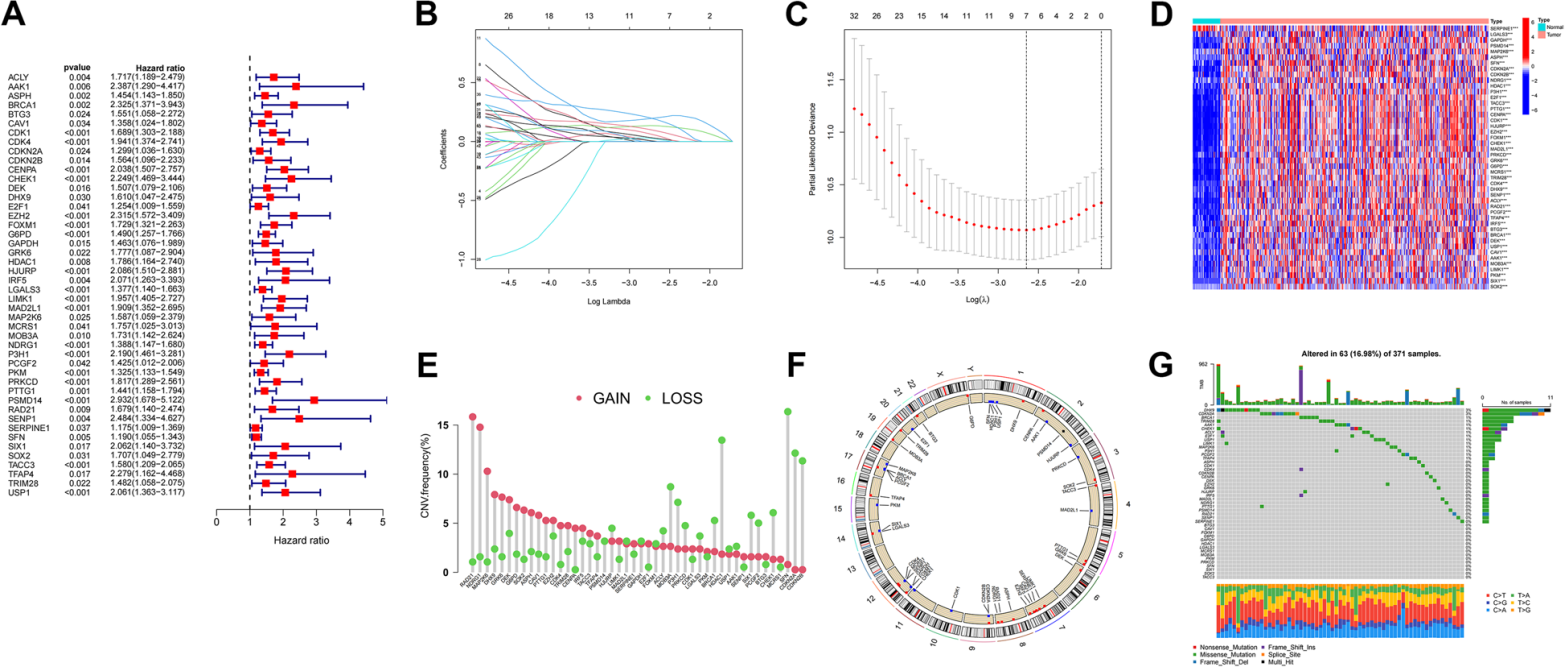
Prognostic characterization of differential genes in cellular senescence. Forest plots, 46 cellular senescence-associated genes associated with HCC prognosis. **A** (LASSO) Cox regression analysis to identify 46 associated senescence differential genes. **B**, **C** Heat map of 46 associated differential senescence-associated genes in cancer and normal tissues of HCC patients. **D** The Bar graph shows the frequency of copy number changes and the Circos plot shows the location of senescence genes in the chromosome, Where red dots indicate gene amplification, blue dots indicate gene deletion, and black dots indicate no significant change. **E**, **F** waterfall plots to show the somatic mutation rate of 46 senescence genes with a mutation frequency of 16.98% **G**

We further assessed the prognostic value of DEGS in HCC patients by univariate Cox regression analysis of the TCGA dataset. 46 cellular senescence DEGs were found to be associated with HCC prognosis by univariate Cox analysis. All 46 senescence-associated genes were significant (*P* < 0.05) in univariate Cox analysis, and high expression was significantly negatively correlated with overall survival of HCC patients (Fig. 3A). Then, patients were divided into training and validation groups. We further analysed these 46 genes by (LASSO) Cox regression 10-fold cross-validation and screened out 7 DEGs associated with HCC prognosis (Fig. 3B, C). Among the screened 46 DEGSs plotted their differential expression heatmap in HCC (Fig. 3D), which demonstrated that these DEGSs were highly expressed in HCC cancers, and the high expression was associated with poor prognosis. Next, we performed copy number variant (CNV) analyses of gain and loss changes in senescence-related genes in HCC patients. As shown in Fig. 3E, RAD21,NDRG1,MAP2K6,DHX9, GRK6 and DEK showed the most extensive CNV gain, while MOB3A,P3H1,HDAC1,SFN,CDKN2A and CDKN2B showed the greatest CNV decrease. Next, we used Circos plots to show the locations of senescence genes in the chromosomes (Fig. 3F). Most senescence-associated genes have fewer or no mutations. We showed the somatic mutation rate of DEGS with a waterfall plot, and the mutation frequency was 16.98%. Among them, DHX9 and CDKN2A had the highest mutation frequency (3%) (Fig. 3G).

### Construction and evaluation of prognostic features

**Fig. 4:**
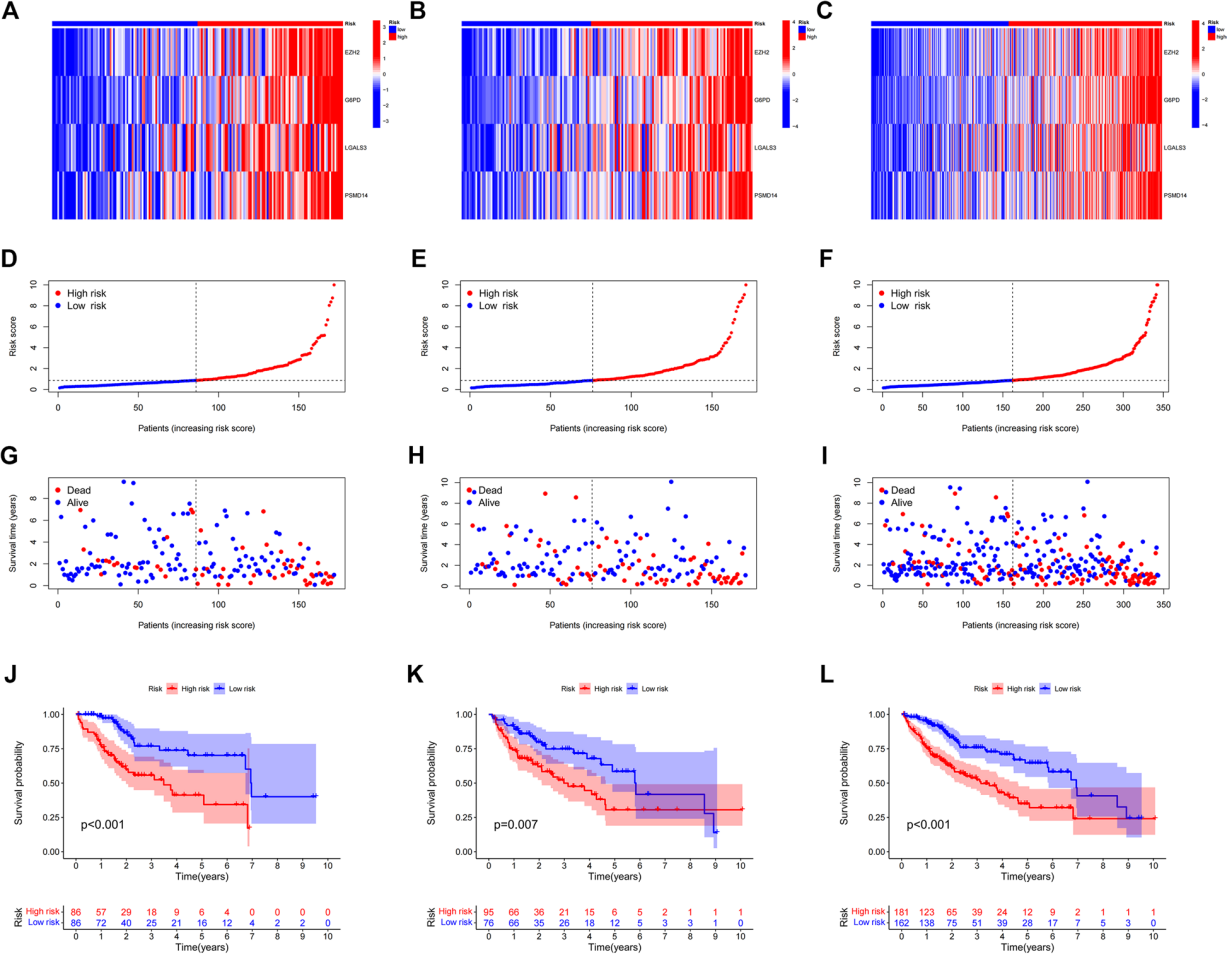
Estimated prognostic models for four senescence-associated genes. Heat map of risk in all HCC samples, EZH2,G6PD,LGALS3,PSMD14 increased with increasing risk score (**A**). Risk heat map of the training set (**B**). Risk heat map of the test set (**C**). Plot of correlation between survival rate and risk score, showed so the risk status of patients with hepatocellular carcinoma (**D**). Correlation plot between training set survival rate and risk score. **E** Correlation plot between test set survival and risk score (**F**). Scatterplot of patient survival in the high and low survival groups of all HCC samples (**G**). Scatterplot of patient survival rates in the high and low survival groups in the training set (**E**). Scatterplot of patient survival in the high and low survival groups in the test set (**F**). Kaplan-Meier survival curves for patients with low risk scores and patients with high risk scores in all HCC patients, Patients with high-risk scores had significantly shorter survival than those with low-risk scores (**J**). Kaplan-Meier survival curve for the training set (**K**). Kaplan-Meier survival curve for the test set (**L**)

Based on the risk score of each HCC patient, patients were divided into high-risk and low-risk groups, and the prognostic models constructed for patients were evaluated using the correlation heat map (Fig. 4A-C), risk score and survival correlation map (Fig. 4D-F) patient survival scatter plot (Fig. 4G-I**)** and Kaplan-Meier survival curves (Fig. 4J-L) for patients in the high-risk and low-risk groups, respectively. In the scatter plot of patient survival versus risk score, survival was significantly correlated with risk score, and the higher the risk score, the lower the survival of patients. The Kaplan-Meier survival curves plotted for the high-risk and low-risk groups showed that patients with high risk scores had significantly shorter survival than those with low risk scores.

### Predicting the prognostic accuracy of patients with HCC

**Fig. 5:**
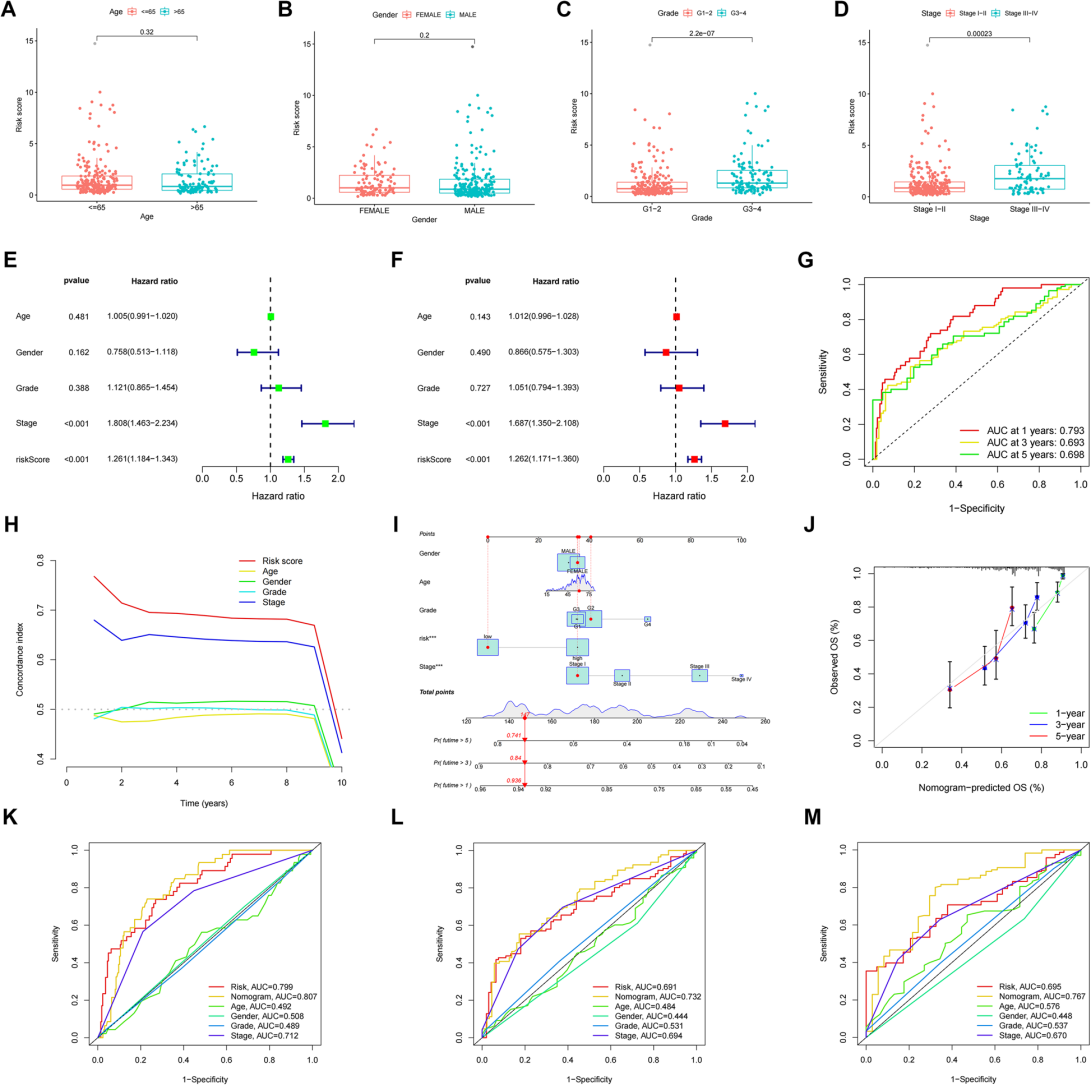
Predicting the prognosis of HCC patients. Risk scores for clinical characteristics (age, gender, class and stage). Age-related risk scores, *P*>0.01 (**A**). Gender-related risk scores, *P*>0.01 (**B**). Grade-related risk scores, *P*<0.001 (**C**). stage-related risk scores, *P*<0.001 (**D**). Univariate Cox regression analysis of the training set., Risk Score, *P*<0.001 (**E**). Multivariate Cox regression analysis of the test set, Risk Score, *P*<0.001 (**F**). ROC curve (AUC) area predicts risk score characteristics for 1-, 3-, and 5-year overall survival.AUC at 1 years:0.793, AUC at 3 years:0.693, AUC at 5 years:0.698 (**G**). C-index graph, The C-index values for risk scores were all higher than the four clinical characteristics of age, gender, grade, and stage (**H**). Nomogram,when total number of HCC patients was 147, the 1-year predicted patient survival rate was greater than 0.936, the 3-year predicted patient survival rate was greater than 0.84, and the 5-year predicted survival rate was greater than 0.741 (**I**). The calibration curves. x-axis is the Nomogram predicted survival and y-axis is the actual survival, and the calibration curves are almost identical to the predicted ones (**J**). Nomogram ROC curve (**K**). Training set Nomogram ROC curve (**L**). Testing set Nomogram ROC curve (**M**)

**Fig. 6:**
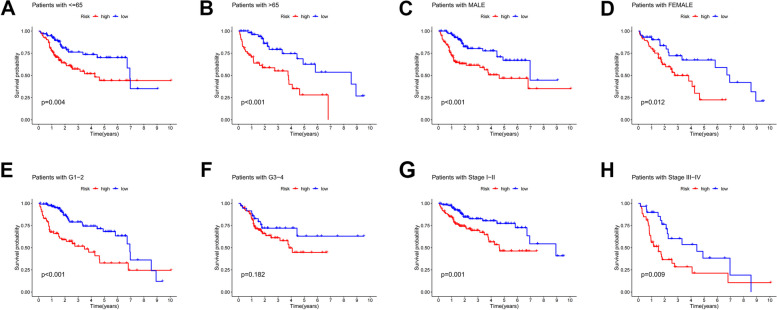
Kaplan-Meier survival curves for different clinical subgroups. including different ages (≤65 and >65), different genders, and different grades and stages. Survival curves for patients ≤65, *P*<0.01 (**A**). Survival curves for patients >65 years of age, *P*<0.001 (**B**). Survival curve of male patients, *P*<0.001 (**C**). Survival curve of female patients, P=0.012 (**D**). Survival curves for grade G1-2 patients, *p*<0.001 (**E**). Survival curves for grade G3-4 patients, *p*=0.182 (**F**). Survival curves for staged I-II patients,P=0.001 (**G**). Survival curves for staged III-IV patients, *P*=0.009 (**H**)

We further validated the accuracy of this feature in predicting the prognosis of HCC patients. We validated the risk differences between the different clinical characteristics in the constructed prognostic models, with significant differences in grade and risk scores (Fig. 5A-D). Similarly, the *p*-values of staged risk scores were less than 0.001 in both univariate and multivariate Cox regression analyses, with significant differences, suggesting that risk scores can predict the prognosis of HCC patients independently of other clinical characteristics (Fig. 5E, F). Based on this feature, the AUC values of patients in the ROC curves at 1-year, 3-year and 5-year survival were all greater than 0.6, which made the prediction more accurate (Fig. 5G). The risk score was a more accurate predictor of prognosis than other clinical characteristics with the highest C-index value (Fig. 5H). Next, we plotted line graphs to predict the survival of HCC patients. When the total number of HCC patients was 147, we predicted a 1-year survival rate greater than 0.936, a 3-year survival rate greater than 0.84, and a 5-year survival rate greater than 0.741 for patients with HCC (Fig. 5I, J). By comparing the calibration curves, we found that the actual 1-year, 3-year, and 5-year survival rates were generally consistent with those predicted, and the maximum area under the ROC curve in the NOMOGRAM plot was 0.807,which demonstrates the accuracy of our model in predicting survival in HCC patients. This suggests that the use of histograms to predict the survival of HCC patients is significantly better than other clinical characteristics (Fig. 5K, L, M), We plotted Kaplan-Meier survival curves for different clinical subgroups. It includes different ages (≤ 65 and > 65 ), different genders, different grades and stages (Fig. 6A-H).

### Functional and pathway enrichment analysis of different senescence-related genes

**Fig. 7:**
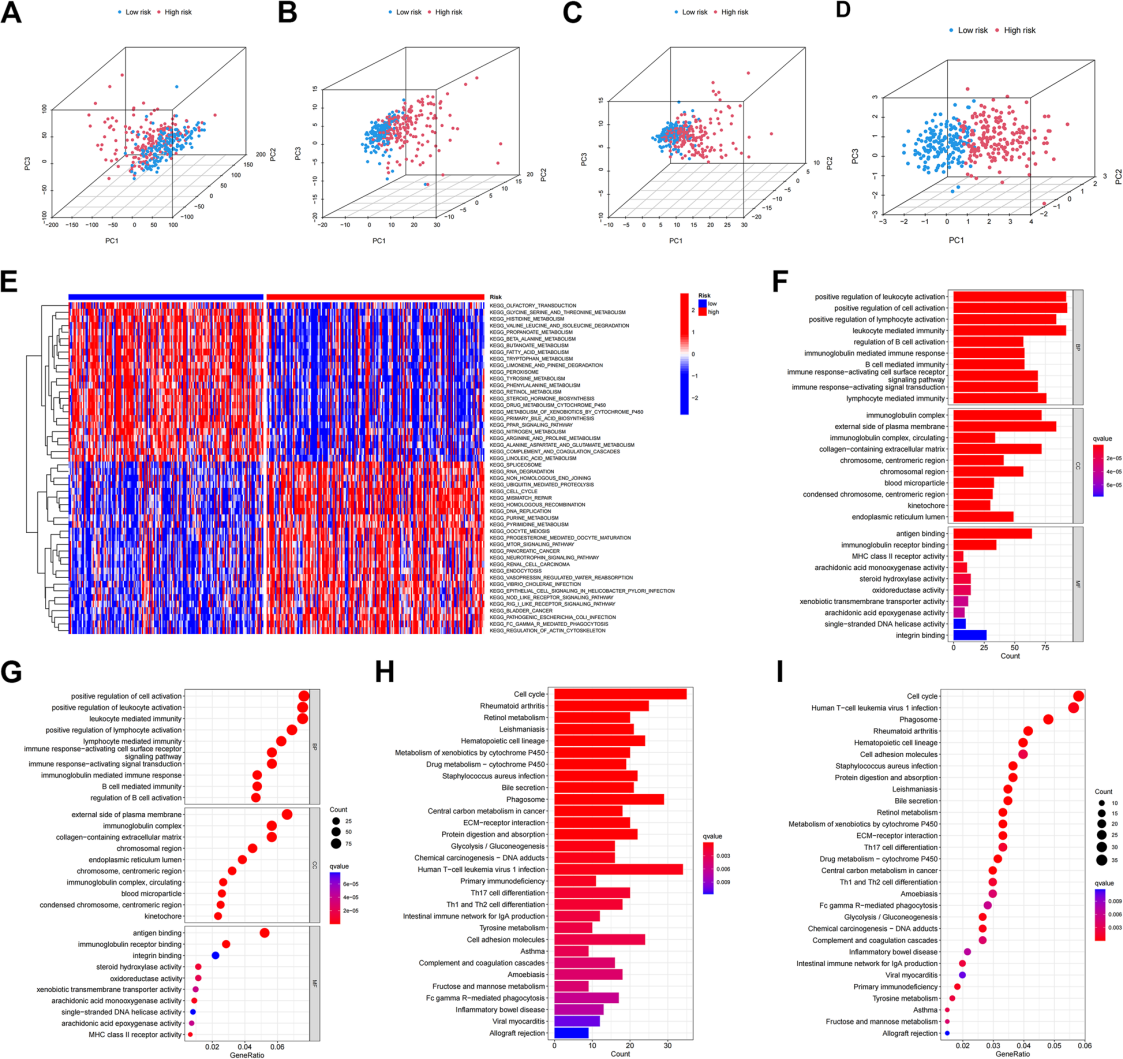
Functional and pathway enrichment analysis of different senescence-related genes. Differences in gene expression distribution throughout (**A**). Differences in the distribution of senescence gene expression (**B**). Differences in the expression distribution of senescence-related genes (**C**). Differences in the distribution of risk model expressions (**D**). Heat map of GSVA enrichment difference analysis between high risk and low risk groups (**E**). GO enrichment in the TCGA cohort. The vertical coordinate is the name of the GO, and the horizontal coordinate is the proportion of the gene (**F**, **G**). KEGG enrichment in the TCGA cohort (**H**, **I**)

PCA was performed to examine the differences in the distribution between the low- and high-risk groups. With these genes, risk genes were more easily separated between high- and low-risk patients (Fig. 7A-D). We next used GO and KEGG pathway enrichment analysis to validate the biological processes (BP), cellular components (CC) and molecular functions (MF) of cellular senescence-associated genes (Fig. 7E-I). We found that differential senescence-associated genes were mainly enriched in leukocyte activation, positive regulation of cell activation, positive regulation of lymphocyte activation, and leukocyte-mediated immunity. In addition, KEGG enrichment analysis was mainly enriched in cell cycle, rheumatoid arthritis, retinol metabolism, leishmaniasis, hematopoietic cell lines, drug metabolism, S. aureus infection, and carbon metabolism in cancer. The most important alterations in the low-risk subgroup were in pathways involving cytokine-cytokine receptor interactions, among others; whereas patients with high-risk scores were mainly focused on tumor-related pathways.

### Immune cell microenvironment and infiltration analysis

**Fig. 8:**
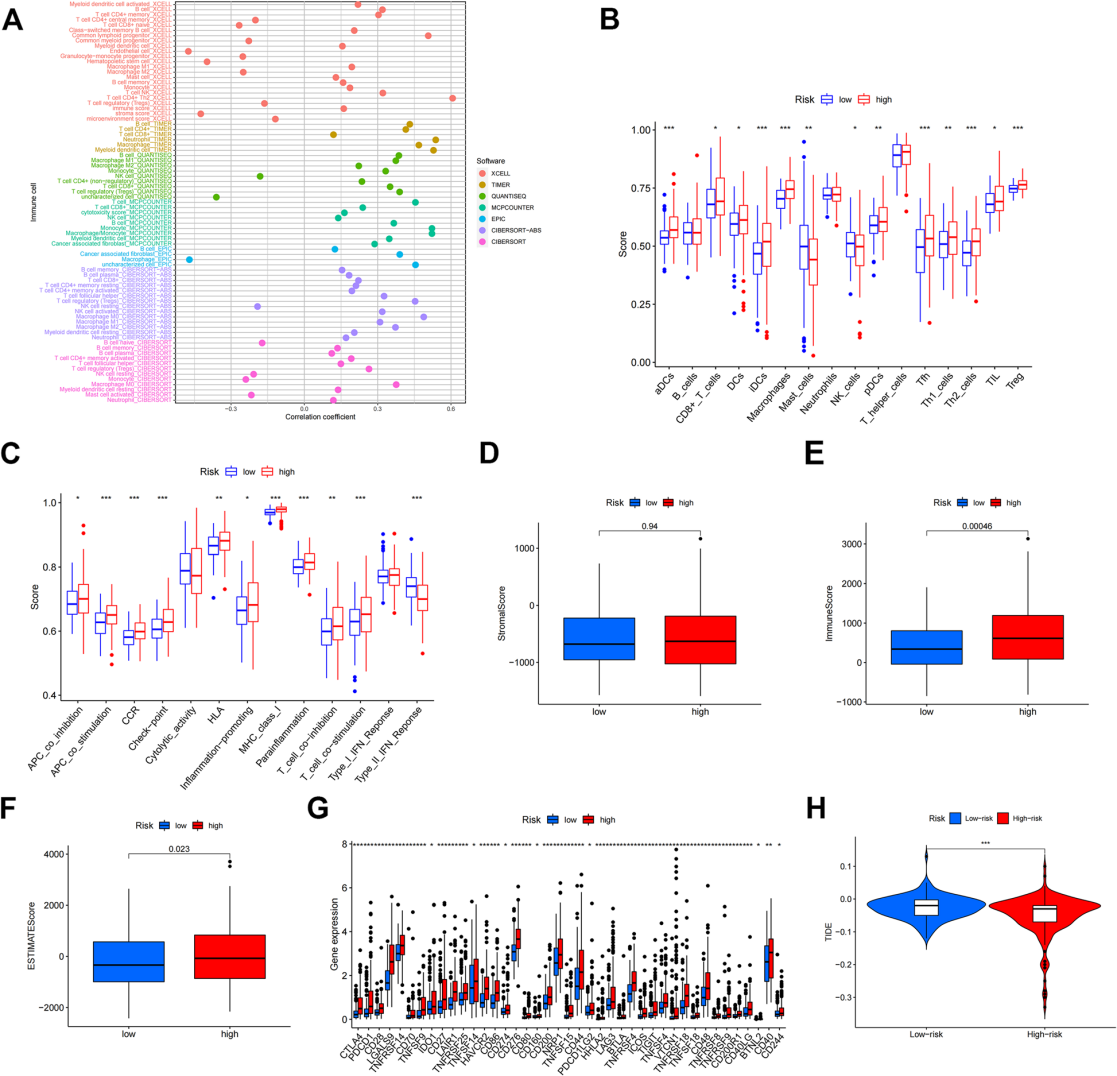
Immune cell bubble plots (**A**). Box plot of the analysis of differences in immune function between high and low risk groups (**B**). Box plots of high and low risk groups in the analysis of differences in risk groups (**C**). Stromal score ,The higher the score, the higher the stromal cell content in the tumor microenvironment (**D**). Immune score, The higher the score, the higher the content of immune cells in the tumor microenvironment (**E**). Estimate score, The higher the score, the higher the content of stromal cells and immune cells in the tumor microenvironment and the lower the content of tumor cells (**F**). Differential expression of 41 immune checkpoint expressions in high and low risk groups (**G**). Violin plot of the analysis of differences in immunotherapy between high and low risk groups (**H**)

Currently, the prognosis of HCC patients depends mainly on cytogenetic and molecular genetic features, but the tumor immune microenvironment also plays a crucial role in the development and course of HCC, and the immune features in the tumor immune microenvironment of HCC are still unknown and need to be further investigated. In the immune cell bubble plots, there was a stronger correlation between various immune cells on different platforms (XCELL, TIMER, QUANTISEQ, MCPCOUNTER, EPIC, CIBERSORT-ABS, and CIBERSORT) (Fig. 8A). The high-risk group, and the degree of immune cell infiltration increased with increasing risk scores in HCC patients. In the ssGSEA analysis, we found that 11 of 16 tumor-infiltrating immune cells were significantly elevated in the high-risk group, and interestingly mast-cells and NK-cells showed a decreasing trend. And 11 of the 13 immunocompetent cells were significantly elevated in the high-risk group. This indicates that the high-risk group has a higher immune infiltration status (Fig. 8B, C). ESTIMATE algorithm is based on single sample Gene Set Enrichment Analysis and generates three scores. In the stromal cell score, immune cell score and composite score: the immune score was higher in the high-risk group and significantly different from the low-risk group, indicating that the tumor microenvironment in the high-risk group had higher levels of stromal cells and immune cells and lower levels of tumor cells (Fig. 8D-F). We then investigated the correlation between the expression of 41 immune checkpoint genes and risk scores (Fig. 8G). High-risk patients exhibited higher expression of immune checkpoint genes. Finally ,we mapped the violin of different risks on immune cell sensitivity (Fig. 8H). Overall, model features affect the tumor-infiltrating immune cell microenvironment and can be potential candidate biomarkers for immunotherapy.

### Relationship between risk score and therapeutic drugs

**Fig. 9:**
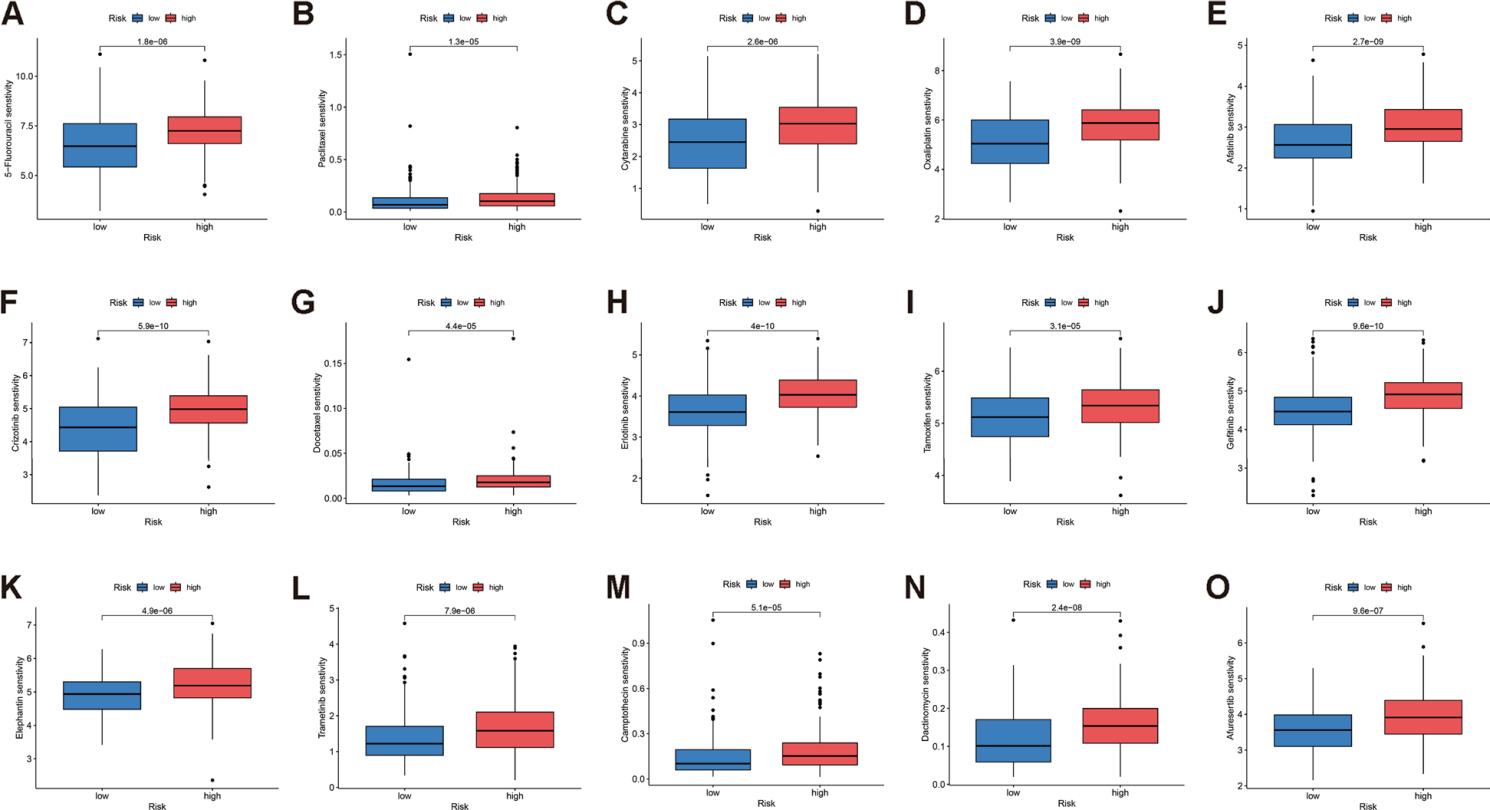
Drug sensitivity analysis of patients with HCC in high and low risk groups (**A**-**O**)

We analyzed the response of HCC patients to chemotherapeutic agents. Compared to patients in the high-risk group, patients in the low-risk group were more sensitive to Fluorouracil, Paclitaxel and Cytarabine., suggests that low-risk patients may respond better to these three chemotherapy drugs (Fig. 9A-C). Low-risk patients were also more sensitive to 12 other drug molecules (Fig. 9D-O).

### Validation of the expression levels of EZH2, G6PD, LGALS3 and PSMD14

**Fig. 10:**
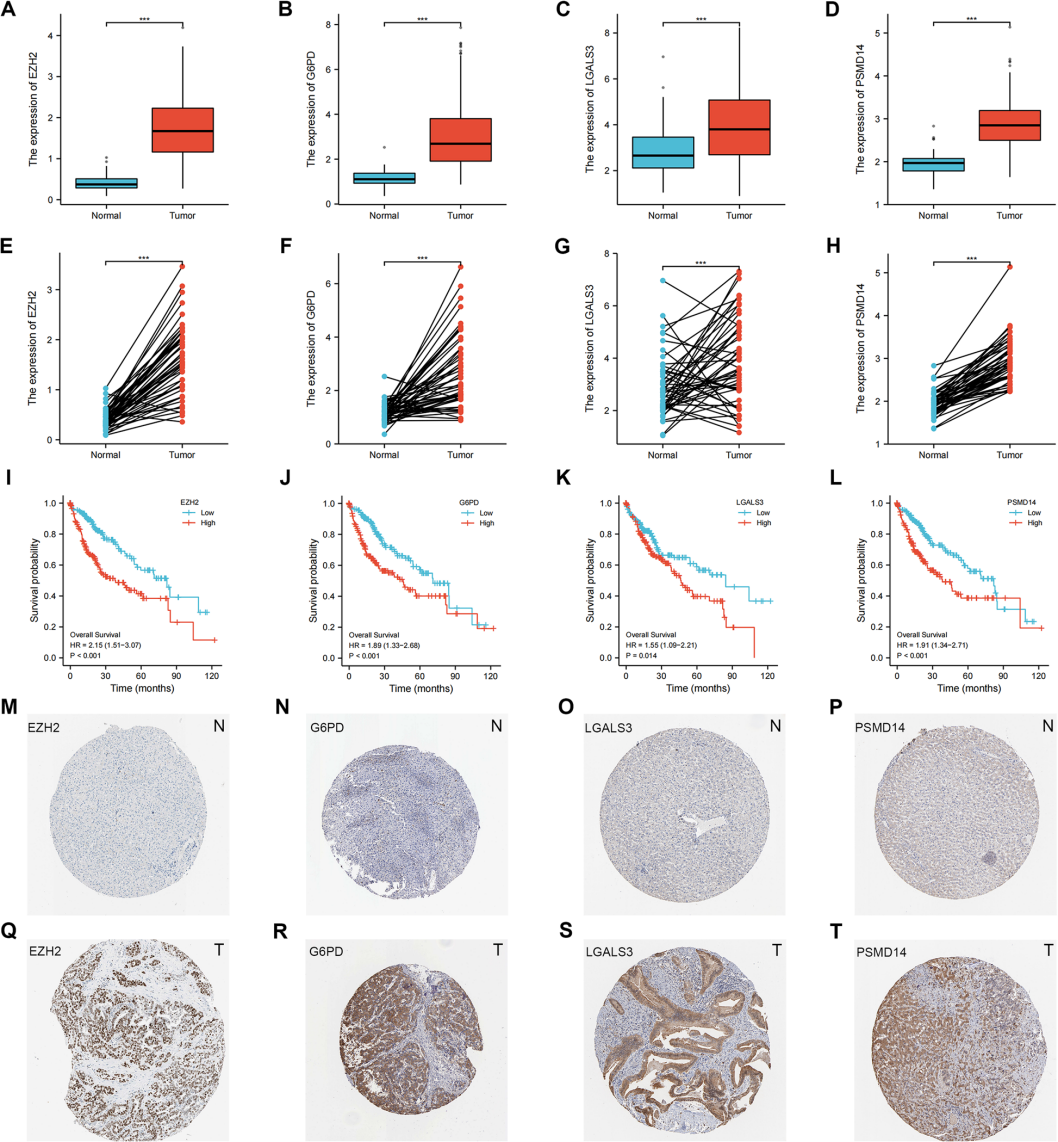
Validation of the expression levels of EZH2, G6PD, LGALS3 and PSMD14. Protein expression levels of EZH2 in unpaired HCC patient samples (**A**). Protein expression levels of G6PD in unpaired HCC patient samples (**B**). Protein expression levels of LGALS3 in unpaired HCC patient samples (**C**). Protein expression levels of PSMD14 in unpaired HCC patient samples (**D**). Protein expression levels of EZH2 in paired HCC patient samples (**E**). Protein expression levels of G6PD in paired HCC patient samples (**F**). Protein expression levels of LGALS3 in paired HCC patient samples (**G**). Protein expression levels of PSMD14 in paired HCC patient samples (**H**). Effect of EZH2 expression on survival of Kaplan-Meyer mapper hepatocellular carcinoma (**I**). Effect of G6PD expression on survival of Kaplan-Meyer mapper hepatocellular carcinoma (**J**). Effect of LGALS3 expression on survival of Kaplan-Meyer mapper hepatocellular carcinoma (**K**). Effect of PSMD14 expression on survival of Kaplan-Meyer mapper hepatocellular carcinoma (**l**). Representative immunohistochemical staining results of EZH2 protein in hepatocellular carcinoma tissue and normal tissue (**M**, **Q**). Representative immunohistochemical staining results of G6PD protein in hepatocellular carcinoma tissue and normal tissue (**N**, **R**). Representative immunohistochemical staining results of LGALS3 protein in hepatocellular carcinoma tissue and normal tissue (**O**, **S**). Representative immunohistochemical staining results of PSMD14 protein in hepatocellular carcinoma tissue and normal tissue (**P**, **T**)

Among the prognostically characterized aging genes, EZH2, G6PD, LGALS3 and PSMD14 were significantly differentially expressed in hepatocellular carcinoma samples from the TCGA database. In both paired and unpaired samples of HCC patients, EZH2, G6PD, LGALS3 and PSMD14 were highly expressed in the cancer and lowly expressed in the paracancer, with statistically significant differences (Fig. 10A-H). High gene expression was significantly correlated with prognosis, with high expression having a significantly poorer prognosis (Fig. 10I-L). We then investigated EZH2, G6PD, LGALS3 and PSMD14 protein levels using the HPA database. The results showed that the protein levels in hepatocellular carcinoma tissues had higher protein levels than the corresponding normal tissues. It suggests that these four senescence-related genes may become potential tumor markers (Fig. 10M-T).

### Validation of EZH2, G6PD, LGALS3 and PSMD14 expression levels in clinical tissue samples by quantitative real-time polymerase chain reaction

**Fig. 11:**
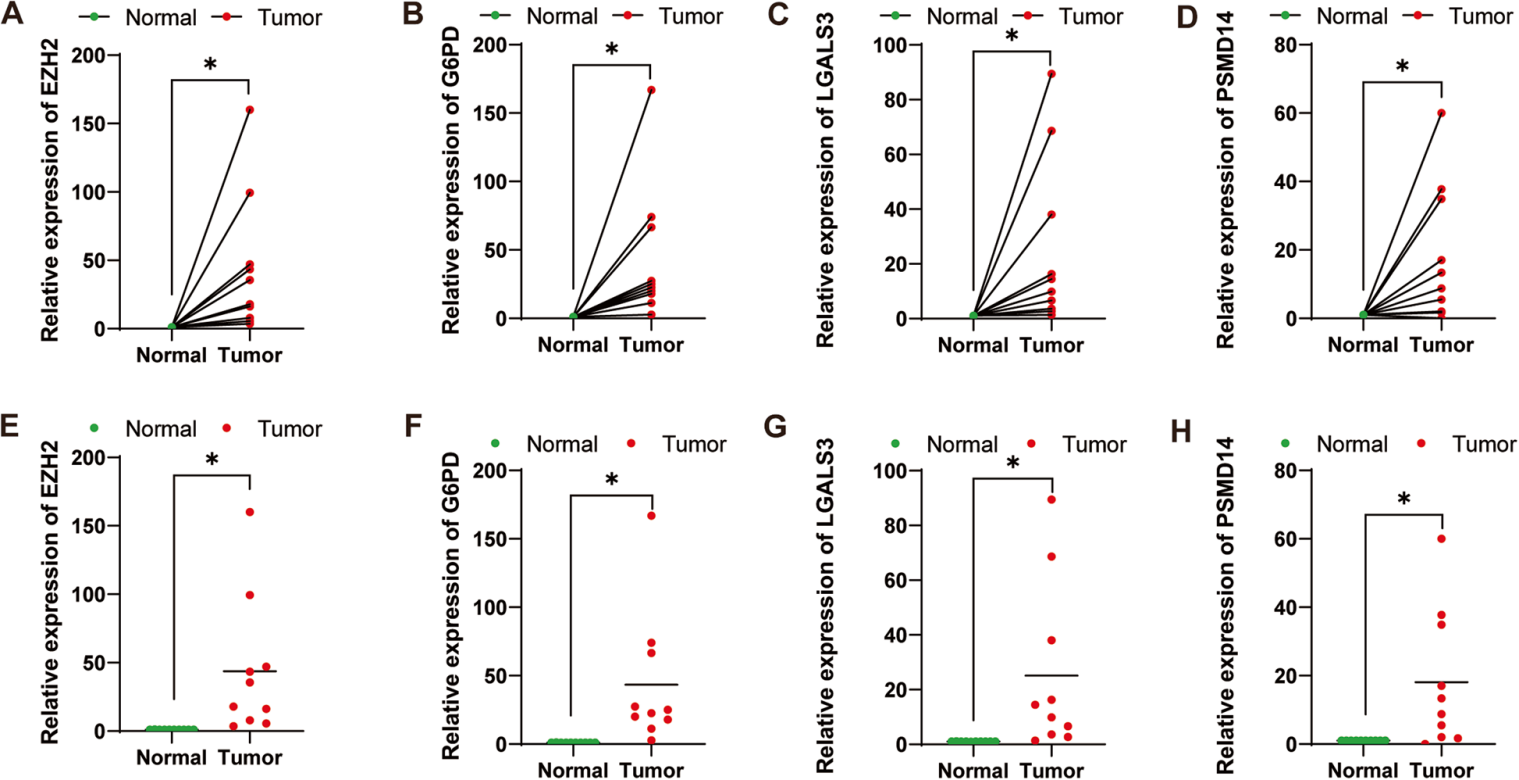
Validation of EZH2, G6PD, LGALS3 and PSMD14 expression levels in clinical tissue samples by quantitative real-time polymerase chain reaction (**A**-**H**)

Among the four genes of prognostic characteristics, the expression levels in hepatocellular carcinoma and adjacent normal tissues were detected by qRT-PCR, EZH2, G6PD, LGALS3 and PSMD14 were all more highly expressed in HCC tumor tissues (*p* < 0.05) (Fig. 11A-H).

## Discussion

Senescence is not determined by a single gene, but is the result of activation and blockage of a cascade of genes and their interaction through their respective products. It prevents the proliferation of cells at risk of tumor transformation and thus acts as a barrier to malignant tumorigenesis in the body [15]. Cellular senescence causes disease development through the senescence-associated secretory phenotype (SASP) [16, 17]. Changes in the regulation of genes and gene interactions during senescence have become the main purpose and direction of research on the mechanisms of senescence [18]. Cellular senescence is a high-risk factor for many diseases, including tumors, and analysis of cellular. It is important to analyze the mechanisms and roles of cellular senescence genes in tumors. In recent years, many studies have constructed prognostic models based on genes associated with different forms of cellular senescence. However, the effects of senescence genes on HCC have not been well investigated.

In our study, we first identified 46 aging-related genes and then identified four pairs of possible prognostic markers by modeling, EZH2, G6PD, LGALS3 and PSMD14.Wu et al. demonstrated that EZH2 is highly expressed in HCC patients and high expression is associated with poor prognosis in HCC patients. The EZH2 gene is associated with immune infiltration in HCC cancer and affects the cell cycle [19]. Zhang et al. proved that EZH2 inhibitors have a long-term anti-proliferative effect in HCC cells and can be a potential therapeutic target by causing sustained reactivation of de novo silenced genes [20]. Wang et al. demonstrated that G6PD was highly expressed in HCC and was associated with pathological staging and poor prognosis, and that G6PD expression was closely related to the immune microenvironment of HCC patients [21]. Song et al. showed that high expression of LGALS3 in HCC cells was closely associated with poor vascular infiltration and survival and enhanced tumorigenesis and metastasis of HCC cells through β-linked protein signaling [22, 23]. Qu et al. demonstrated that LGALS3 expression was positively correlated with highly sensitive anticancer drugs in HCC, respectively, which provides a potential target for HCC treatment [24]. Lv et al. proved that PSMD14 was significantly upregulated in the tissues of HCC patients. and that PSMD14 overexpression was related with vascular infiltration, tumor metastasis, tumor recurrence, and survival. PSMD14 can promote phenotypic changes in HCC cells in vitro, including affecting cell proliferation invasion and migration. [25]. We divided all HCC patients from TCGA and GEO databases into two clusters of HCC samples based on cellular senescence-associated gene expression. Cluster A had a significantly better prognosis than cluster B. Not only in HCC patients, these four genes have been found to be associated with various other types of malignancies. High levels of EZH2 are not only associated with breast cancer tumor immune response [26], Ezh2 also acts with Stat3 as a transcriptional activator to alter the tumor immune response in melanoma [27]. G6PD is highly expressed in renal cell carcinoma, and high expression affects the cell cycle [28]. LGALS3 has been considered as one of the promising targets for cancer immunotherapy, and LGALS3 inhibitors can inhibit T cell proliferation and activation through ligand combinations [28]. PSMD14 is highly expressed in esophageal cancer in esophageal cancer, and inhibition of PSMD14 inhibits tumor cell migration, invasion and EMT [29]. These results suggest that these four genes could be potential biomarkers for cancer treatment diagnosis and healing.

In the validation of the unused database dataset, we all found that the survival rates of the constructed prognostic models were better in the low-risk group than in the high-risk group.We next verified that risk score and tumor grading characteristics can be prognostic factors for HCC patients by LASSO, univariate and multifactorial COX analyses. The KM curves also provide side-by-side evidence that survival is better in low risk scores than in patients with high risk scores. We then used ROC analysis to verify the accuracy of the predictions and to make validity evaluations of patients’ 1-year, 2-year, and 3-year survival. We then plotted column line plots of patient survival probabilities, which are based on multifactorial regression analysis, integrating multiple predictors and using graphs to derive patient survival probabilities. The calibration curve shows that the predicted and actual occurrence rates are close and the model is well calibrated. Finally, the ROC areas of the risk curves under the Nomogram model were all higher than other clinical characteristics, indicating that the Nomogram model has higher accuracy in predicting survival of HCC patients, which was laterally verified by the calibration curves. Meanwhile, KEGG enrichment analysis was mainly enriched in cell cycle and tumour-related pathways [30–32].

Currently, there is a paucity of effective drugs for the treatment of HCC patients, therefore, in-depth study of drug sensitivity in HCC patients is important for the development and optimization of targeted therapies for hepatocellular carcinoma. We investigated the sensitivity of different risk groups of the model construct to chemotherapeutic drugs, and the low-risk group may be more sensitive to common chemotherapeutic drugs and molecularly targeted drugs.The use of risk scores can be individualized for chemotherapy of HCC patients. With the prolongation of chemotherapy, tumors tend to develop drug resistance and are prone to recurrence. On this basis, tumor immunotherapy is developing rapidly [33–36]. However, the liver is considered an immune-tolerant tissue, a characteristic that can be attributed to the specificity of its physiological function. Among the new developments are studies suggesting that TGF-beta will contribute to immunotherapy in HCC [37–39]. TGF-β affects the immune microenvironment by influencing HCC infiltrating CD8 T cells [40]. he identification of new immune markers remains a major goal of HCC research. In our study, the level of immune infiltration was higher in the low-risk scoring group. Regulatory T-cell infiltration was higher in the high-risk scoring group, whereas NK-cell and mast cell follicular helper infiltration was higher in the low-risk scoring group. The immune function analysis also showed that “MHC_class_I” was higher in patients with high risk scores. In the low-risk group, “cytolytic_activity” and “type_II_IFN_response” were more active. “MHC_class_I” is a tissue-specific proteasome produced by the thymus proteasome and immune proteasome [41]. NK cells can produce inflammatory cytokines and chemokines that limit tumor growth by lysing transformed cells, but the functional activity of NK cells is inhibited by MHC molecules displayed on these cells, while NK cells can enhance the activity of tumors that lose MHC class I expression [42, 43]. This is exactly in line with what we found, and it is our next research direction to make NK cells with therapeutic effect in the hypofractionated risk group to play a greater potential role as a new weapon for cancer therapy and to play a greater role. Our findings can be used to guide clinical chemotherapy dosing and immunotherapy in HCC patients. We finally verified the mRNA expression of EZH2, G6PD, LGALS3 and PSMD14 by qRT-PCR in clinical gastric cancer tissue samples. The results showed that mRNA expression of EZH2, G6PD, LGALS3 and PSMD14 was elevated in cancer tissues from HCC patients compared with normal gastric cancer parietal tissues, and the difference was statistically significant in the parietal cancer, which may suggest EZH2, G6PD, LGALS3 and PSMD14 as potential biomolecular markers for HCC.However, there are some shortcomings in our study. Our samples were obtained from public databases and all samples were obtained retrospectively, prospective cohort studies are still necessary. Furthermore, in addition to internal validation we need to add external validation, the study was performed with limited qRT-PCR. experiments and further cellular experiments, animal experiments and clinical cases are needed to validate our experimental results.

Overall, the construction of a prognostic model for cellular senescence risk score can not only be used to predict the survival of HCC patients, but also give more strategies for the clinical treatment of HCC patients. However, this study has some limitations, and more cytological experiments, animal experiments and clinical data are needed to validate the novel molecular mechanisms of model modulation of gastric cancer phenotype.

### Supplementary Information

**Additional file 1.**

**Additional file 2.**

## Data Availability

Datasets generated and analyzed during the current study were downloaded from TCGA (https://portal.gdc.cancer.gov) and cellular senescence-associated genes were downloaded from CellAge (https://genomics.senescence.info).
