# Supplementary material for: Cellular senescence-related genes: predicting prognosis in hepatocellular carcinoma
Source: BMC Cancer. 2023 Oct 18;23:1001. doi: 10.1186/s12885-023-11288-1 (PMC10585749; doi:10.1186/s12885-023-11288-1)
Supplement: Supplementary file 2 — Additional file 2. [file 12885_2023_11288_MOESM2_ESM.pdf]

Table S2: Primer sequences used in this study

EZH2
Forward SequenceGACCTCTGTCTTACTTGTGGAGC
Reverse Sequence CGTCAGATGGTGCCAGCAATAG

G6PD
Forward SequenceCTGTTCCGTGAGGACCAGATCT
Reverse Sequence TGAAGGTGAGGATAACGCAGGC

LGALS3
Forward SequenceCCATCTTCTGGACAGCCAAGTG
Reverse Sequence TATCAGCATGCGAGGCACCACT

PSMD14
Forward SequenceGTCAGTGTGGAGGCAGTTGATC
Reverse Sequence CCACACCAGAAAGCCAACAACC
